# Comparison of the Effects of Ringer’s Lactate and 6% Hydroxyethyl Starch 130/0.4 on Blood Loss and Need for Blood Transfusion After Off-Pump Coronary Artery Bypass Graft Cardiac Surgery

**DOI:** 10.7759/cureus.16049

**Published:** 2021-06-30

**Authors:** Roshani Manwani, Neha Gupta, Shilpa Kanakam, Minal Vora, Krishnan Bhaskaran

**Affiliations:** 1 Anesthesiology, Dr. Dnyandeo Yashwantrao (DY) Patil University, School of Medicine, Navi Mumbai, IND; 2 Anesthesiology, Dr. Dnyandeo Yashwantrao (DY) Patil Medical College and Hospital, Navi Mumbai, IND; 3 Anesthesiology, Fortis Hospital, Kolkata, IND; 4 Anesthesiology, Apollo Hospitals, Chennai, IND

**Keywords:** blood loss, fluid regimen, coagulation, cardiac surgery, crystalloid, colloid

## Abstract

Background

Infusion of crystalloids fluid replacement therapy tends to cause a greater expansion of intravascular volume. However, colloids can affect blood coagulation leading to greater blood loss and transfusion requirements. This study compared the intraoperative and postoperative blood loss with Ringer’s lactate (RL) versus 6% hydroxyethyl starch (HES) 130/0.4 as infusion fluid during cardiac surgery.

Methods

Eighty adult male and female patients undergoing elective cardiac surgery were randomly assigned to receive either RL or 6% HES 130/0.4 20 ml/kg during off-pump coronary artery bypass graft (OP-CABG) surgery. Intraoperative blood loss and 24 hours postoperative chest tube drainage were the primary outcomes. Simultaneously, blood transfusions, thromboelastometry variables, total fluid requirement, renal function, and intensive care unit (ICU) stay were assessed.

Results

The intraoperative blood loss was similar (p > 0.05) with HES (716 ml) and RL (658 ml). Postoperative chest tube drainage was higher (p < 0.05) with HES (513 ml) as against RL (449 ml). The total fluid requirement was higher in the RL group. Alteration of thromboelastometry variables, renal function, and ICU stay was comparable between the two groups. Postoperative chest tube drainage was less with the use of RL during cardiac surgery. A lesser total fluid requirement in the HES group did not lead to any improvement in renal function and the length of ICU stay.

Conclusions

Crystalloids (RL) provide similar outcomes to HES and can be used as substitutes to colloids during cardiac surgery. However, further large-scale multicenter studies with varied indications can be suggested to substantiate the equivalence of crystalloids to colloids in perioperative management.

## Introduction

Patients with coronary artery bypass graft (CABG) surgery may be hemodynamically unstable during the immediate postoperative period and need fluid support [1]. Colloids such as hydroxyethyl starches are known to have a more profound intravascular volume expansion compared with crystalloids such as Ringer’s lactate (RL) [2]. Hence, ideally, colloids should be suitable for restrictive fluid therapy during any major surgery. However, a lot of controversies still exist about the optimal perioperative fluid management in patients undergoing cardiac surgery, especially due to their effects such as inflammatory responses, effects on endothelial integrity, and effects on organs such as the kidney [3].

Five percent human albumin (5% HA) has been in use for a long time during cardiac surgery as a colloid. However, due to its cost and availability, colloids such as hydroxyethyl starches (HES) are being used. However, HES preparations have shown to impair coagulation and renal function [4,5]. HES has been reported to cause mild systemic acidosis in patients undergoing normovolemic hemodilution after cardiac surgery and impairment in fibrin formation and clot strength [3,6]. Also, correcting hypovolemia with HES has been suggested to be associated with an increased risk of acute renal failure [7]. Six percent HES (6% HES 130/0.4) is a newer generation tetra starch formulation with a lower molecular weight, which might affect coagulation to a lesser degree [8]. Although cheaper than HA, 6% HES 130/0.4 is 4-10 fold dearer than crystalloids in India. Transfusion of packed red blood cells (PRBCs) fluid replacement is associated with increased morbidity and mortality after cardiac surgery [9]. RL has also been used for many years during heart surgery, either as a sole replacement fluid or in combination with colloids [10]. Because of the large volumes administered throughout the procedure, even RL might influence coagulation via dilution of coagulation factors. Impaired coagulation leads to higher perioperative blood loss, leading to a higher requirement of blood transfusion.

The aim of this study was to compare the effect of 6% HES solution with RL solution on intraoperative and postoperative blood loss when used as the main infusion during cardiac surgery in patients after CABG surgery.

## Materials and methods

Study participants

Patients posted for coronary artery bypass graft (CABG) surgery at the study sites were screened for eligibility based on the study inclusion/exclusion criteria. The study documents were reviewed and approved by the Institutional Ethics Committee (IEC). Male and female patients between 30 and 70 years of age who were planned to undergo elective off-pump (OP) CABG and those who signed the informed consent were included. Patients with known allergy to HES, presence of anemia, coagulation disorders (INR > 1.2), activated partial thromboplastin time > 40 seconds, platelet count < 0.5 million cells/cubic mm), and left ventricular ejection fraction (LVEF) < 40% were not included. Also, those with serum creatinine > 1.5 mg/dl or those who are receiving any anticoagulants or antiplatelet therapy within seven days before surgery were excluded. A total of 98 patients were screened out of which 80 were enrolled and randomized in this randomized, comparative, double-blind study.

Randomization, blinding, and fluid regimen

Patients were randomized to receive either RL (n = 40) 20 ml/kg body weight or HES (n = 40) 20 ml/kg body weight. Randomization was performed using the sealed envelope technique. The hospital pharmacy prepared the study solutions that were supplied in identical 500 ml bottles. Bottles and infusion sets were wrapped with opaque covers for blinding. Normal saline (0.9%) was used as fluid for any additional requirement.

Procedures

Anesthesia was induced with thiopental (5 mg/kg) and fentanyl (5 mcg/kg), and tracheal intubation was facilitated by rocuronium (0.6 mg/kg) intravenously. Propofol (1 mg/kg/hr) and fentanyl (2 mcg/kg/hr) infusions were used for maintenance. Muscle paralysis was maintained with rocuronium infusion (0.3 mg/kg/hr).

Fluid administration was started with 250-500 ml of study solution during induction of anesthesia. The remaining allotment of the study solution was used subsequently. Any additional fluid requirement was fulfilled with 0.9% normal saline (NS) in both groups. Baseline thromboelastometry variables were measured after induction of anesthesia. All patients received the same standard dose of heparin and tranexamic acid. Activated clotting time (ACT) was measured regularly and maintained above 300 seconds till the completion of the anastomosis. After the procedure, the heparin effect was reversed using protamine (1.5 mg for each 100 IU of heparin given). Norepinephrine was used, if necessary, to maintain mean arterial pressure > 60 mm Hg. Transoesophageal echocardiography was used to assess ventricular function. Intraoperative blood loss was measured by measuring blood in the suction bottle and mops (by gravimetric method). PRBCs were transfused when hemoglobin (Hb) was less than 7 gm%. The administration of fresh-frozen plasma (FFP), platelets, and coagulation factors was based predominantly on thromboelastometry variables and the pre-operative and postoperative coagulation profile of each patient.

After the procedure, the patient was transferred to the intensive care unit (ICU), and further fluid management and inotrope support were at the discretion of the attending consultant and not controlled by protocol. Postoperatively, the amount of chest drains for 24 hours and blood transfusion requirements were measured. Blood samples were collected to assess complete blood counts, renal function tests, and thromboelastometry 24 hours after surgery.

Statistical analysis

All the continuous variables were assessed for normality using Shapiro-Wilk test. Normally distributed data are presented as means with standard deviation (SD), whereas non-normal data are presented as medians with interquartile range (IQR). All the categorical variables were expressed either as percent or proportion. Comparison of normally distributed continuous variables was done by independent sample test or analysis of variance (ANOVA). Comparison of non-normally distributed continuous variables was done by Mann-Whitney U test or Kruskal-Wallis H test.

Comparison of categorical variables was done by Chi-square test or Fisher’s exact test based on the number of observations. Data entry was done in Microsoft Excel (Microsoft Corporation, New Mexico, USA), and data analysis was carried out using Statistical Package for the Social Sciences (SPSS) version 17.0 (SPSS Inc., Chicago, USA). All the p-values < 0.05 were considered statistically significant.

## Results

**Figure 1:**
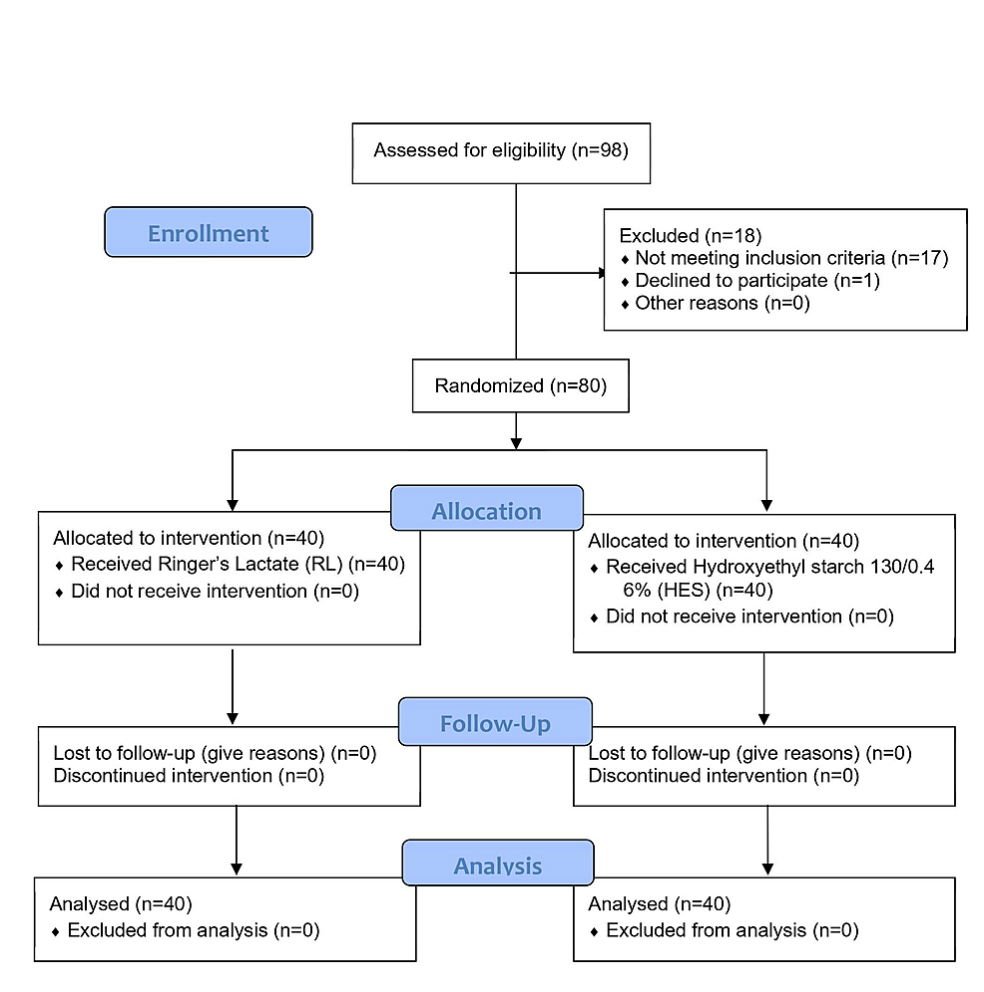
CONSORT 2010 flow diagram

All randomized patients completed the study as per the protocol. Figure 1 shows the CONSORT flow chart for the patient in the study. The two groups were similar with respect to the demography and baseline characteristics (Table 1).

 

**Table 1:** Demography of the randomized patients RL: Ringer lactate; HES: hexaethyl starch; SD: standard deviation; CI: confidence intervals.

	RL (n = 40)	HES (n = 40)	Independent Sample t-Test			
	Mean (SD)	Mean (SD)	t	p	Mean diff.	(95% C.I.)
Age (yrs)	46.13 (6.69)	47.35 (6.67)	-0.820	0.415	-1.220	(-4.20–1.75)
Weight (kg)	68.4 (8.07)	66.24 (9.76)	1.078	0.285	2.16	(-1.83–6.14)
Height (m)	1.64 (0.08)	1.63 (0.09)	0.918	0.362	0.02	(-0.02–0.05)
BMI (kg/m^2^)	25.3 (1.88)	24.92 (2.32)	0.799	0.427	0.38	(-0.56–1.32)
INR (pre-operative)	1.01 (0.04)	1.02 (0.06)	-0.941	0.349	-0.01	(-0.03–0.01)
	No. (%)	No. (%)		P*		
Gender						
Male	37 (92.50%)	34 (85.00%)		0.481		
Female	3 (7.50%)	6 (15.00%)				
*Fisher’s test						

The pre-operative data, postoperative data, and the change from pre-operative data are presented in Table 2 for the different parameters assessed for the study.

**Table 2:** Comparison of laboratory parameters in two groups RL: Ringer lactate; HES: hexaethyl starch; SD: standard deviation; CI: confidence intervals.

	RL (n = 40)	HES (n = 40)	Independent Sample t-Test			
	Mean (SD)	Mean (SD)	t	p	Mean diff.	(95% C.I.)
Hematocrit (%)						
Pre-operative	38.5 (3.23)	39.28 (1.8)	-1.327	0.188	-0.77	(-1.94–0.39)
Postoperative	31.63 (3.45)	31.18 (2.49)	0.668	0.506	0.45	(-0.89–1.79)
Change	-6.88 (3.78)	-8.1 (2.84)	1.638	0.105	1.23	(-0.26–2.71)
Platelets (X0.1 million cell/cmm)						
Pre-operative	2.50 (0.64)	2.36 (0.6)	0.981	0.329	0.14	(-0.14–0.41)
Postoperative	2.19 (0.53)	2.00 (0.62)	1.503	0.137	0.19	(-0.06–0.45)
Change	-0.31 (0.41)	-0.37 (0.5)	0.587	0.559	0.06	(-0.14–0.26)
Serum creatinine (mg/dl)						
Pre-operative	0.94 (0.15)	0.97 (0.17)	-1.054	0.295	-0.04	(-0.11–0.03)
Postoperative	0.89 (0.16)	0.99 (0.21)	-2.358	0.021	-0.10	(-0.18–-0.02)
Change	-0.05 (0.14)	0.01 (0.2)	-1.663	0.100	-0.06	(-0.14–0.01)
Reaction time R (mm)						
Pre-operative	6.34 (2.3)	7.20 (2.53)	-1.580	0.118	-0.86	(-1.93–0.22)
Postoperative	4.96 (1.69)	5.66 (1.77)	-1.816	0.073	-0.70	(-1.47–0.07)
Change	-1.39 (2.98)	-1.54 (2.92)	0.231	0.818	0.15	(-1.16–1.47)
Clot formation time K (mm)						
Pre-operative	1.78 (0.61)	1.92 (0.51)	-1.135	0.260	-0.14	(-0.39–0.11)
Postoperative	1.47 (0.45)	1.72 (0.66)	-1.960	0.054	-0.25	(-0.50–0.00)
Change	-0.31 (0.8)	-0.20 (0.8)	-0.588	0.558	-0.11	(-0.46–0.25)
Alpha angle						
Pre-operative	64.60 (7.35)	61.47 (8.35)	1.780	0.079	3.13	(-0.37–6.63)
Postoperative	68.18 (6.64)	65.65 (8.31)	1.501	0.137	2.53	(-0.82–5.87)
Change	3.58 (9.48)	4.19 (10.07)	-0.277	0.783	-0.61	(-4.96–3.75)
Maximum amplitude						
Pre-operative	69.81 (5.61)	67.95 (5.41)	1.505	0.136	1.86	(-0.60–4.31)
Postoperative	72.35 (4.65)	70.56 (5.22)	1.619	0.109	1.79	(-0.41–3.99)
Change	2.54 (5.77)	2.61 (6.82)	-0.046	0.963	-0.07	(-2.88–2.75)

**Figure 2:**
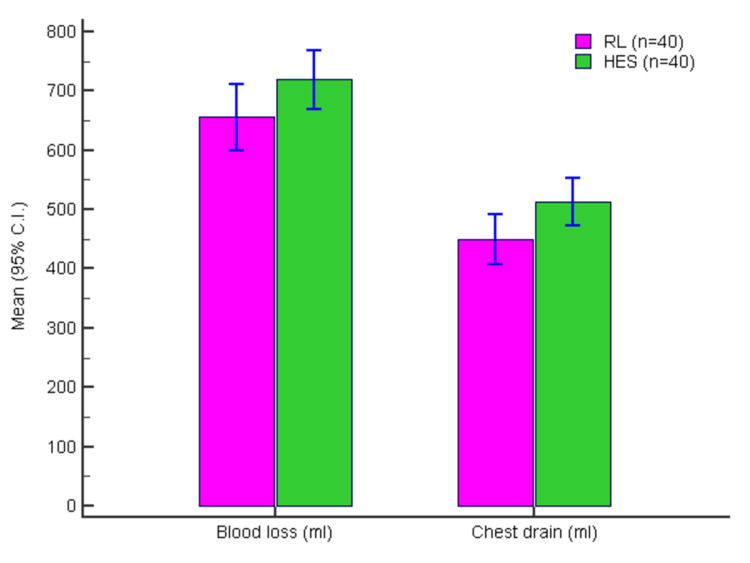
Intraoperative blood loss (ml) and chest drain volume (ml) in two groups RL: Ringer lactate; HES: hexaethyl starch.

Figure 2 presents the intraoperative blood loss with HES and RL.

**Figure 3:**
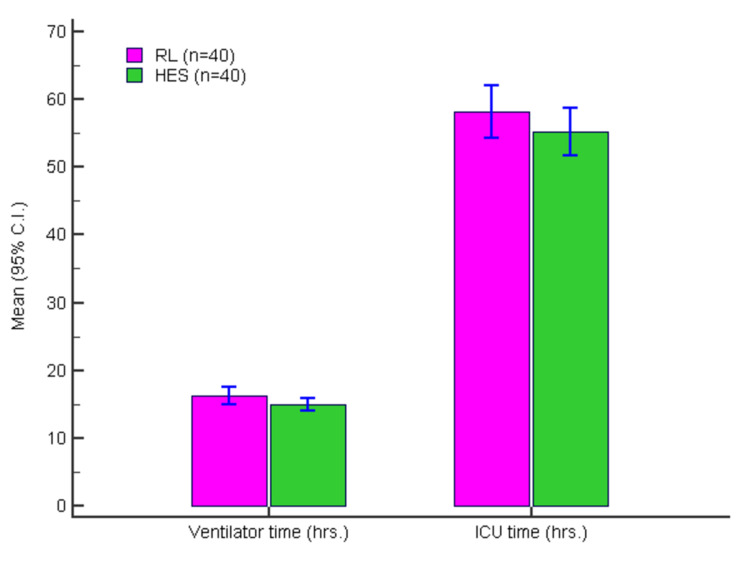
Duration of ventilator use (hrs) and ICU stay (hrs) in two groups RL: Ringer lactate; HES: hexaethyl starch; ICU: intensive care unit.

Figure 3 presents the duration of ventilator use (hours) and the ICU stay (hours).

There were no differences between the HES and RL groups (adjusted for covariates) for change in laboratory parameters (Table 3). This signifies a similar safety profile of HES and RL.

**Table 3:** Unadjusted and adjusted means (ANCOVA) for change in laboratory parameters in two groups * Adjusted for multiple comparisons with age, gender, BMI, and need for PRBC as covariates using ANCOVA. PRBS: Packed red blood cells; BMI: body mass index; ANCOVA: multiple analysis of covariance.

	Unadjusted Mean Difference	Adjusted Mean Difference*	p	95% C.I. for the Adjusted Mean Difference
Hematocrit (%)	1.225	1.292	0.089	(-0.20–2.78)
Platelets (lakh/cmm)	0.060	0.044	0.663	(-0.16–0.24)
Serum creatinine (mg/dl)	-0.063	-0.072	0.064	(-0.15–0.00)
Reaction time R (mm)	0.153	0.059	0.932	(-1.31–1.42)
Clot formation time K (mm)	-0.105	-0.062	0.737	(-0.43–0.31)
Alpha angle	-0.605	-0.746	0.741	(-5.23–3.73)
Maximum amplitude	-0.065	0.031	0.983	(-2.85–2.91)

## Discussion

Optimization of perioperative fluid therapy helps in reducing the morbidity and mortality associated with CABG. The type of fluid to be used is still controversial. Colloids have the theoretical advantage of greater retention in the intravascular compartment, thereby reducing the fluid requirement. However, a high cost and higher chances of coagulopathy and renal injury limit its use. The various side effects associated with first and second degree HES such as coagulopathy, anaphylactoid reactions, renal impairment, and increase in serum amylase levels led to development of newer starch-based volume expanders like 6% hydroxyethyl starch 130/0.4 [11]. The molecular weight and degree of substitution may be mainly responsible for varying effects of different HES solutions on hemostasis [4]. However, there are very few studies that directly compare the effect on coagulation between 6% hydroxyethyl starch 130/0.4 and crystalloids. An in-vitro study by Casutt et al. (2010) found fewer effects on blood coagulation using crystalloids than colloids [12]. Skhirtladze et al. (2014) found greater hemodilution and interference with coagulation with colloids when compared with crystalloids leading to greater need for blood transfusion [10]. In another prospective case-control study involving 50 CABG patients receiving acute normovolemic hemodilution (ANH), there were no significant changes in coagulation state with saline solution (SS) [13]. However, Ruttmann et al. (2002) reported rapid pre-operative hemodilution with crystalloids leading to enhanced coagulation as against no enhancement of coagulation with colloids [14]. Low molecular weight-hydroxyethyl starch (LMW-HES) 130/0.4 was better than medium molecular weight-hydroxyethyl starch (MMW-HES) 200/0.5 and gelatin in patients undergoing off-pump (OP)-CABG, in terms of better preservation of coagulation associated with enhanced volume effect [15]. In a randomized, double-blind clinical trial, HES (6%) had a better volume-expanding effect than gelatin (4%) and Ringer's solutions, and its short-term effects on renal function were also better than gelatin and Ringer's solutions [16]. In a prospective, randomized clinical trial, 132 patients undergoing CABG, administration of tranexamic acid in HES 130/0.4 prime solution study group decreased the estimated blood loss and chest tube drainage in comparison to patients receiving Ringer prime solution with or without tranexamic acid postoperatively [17]. In a randomized, controlled study similar to ours involving adults after on-pump CABG procedures, there was no significant difference in blood loss or blood coagulation between the 130/0.4 (HES 6%) group and the gelatin group [18].

We compared the effect of RL solution versus HES (130/0.4) 6% solution on intraoperative and postoperative blood loss when used as the main infusion during cardiac surgery in patients after CABG surgery. In our study, there was no significant difference in the extent of hemodilution seen in the two groups. The postoperative chest tube drainage was higher in the HES group, but that did not lead to any significant increase in the need for blood transfusion. This can be explained by the lower cut-off hemoglobin of 7% for transfusing blood, which we followed in the study. The reduction in total fluid requirement in the HES group did not translate into any reduction in the ICU and overall hospital length of stay. Alteration of coagulation and renal function assessed by thromboelastography (TEG), serum creatinine, and urine output was also comparable in the two groups.

The concentration of intravenous (IV) solution influences the initial volume effect, i.e., 6% HES solutions is iso-oncotic in vivo, with 1 L replacing about 1 L of blood loss, whereas 10% solutions are hyperoncotic, with a volume effect considerably exceeding the infused volume (about 145%). The HES (130/0.4) 6% is a low molecular weight third-generation starch prepared to have minimal effects on the coagulation, kidney, and serum amylase levels apart from being less immunogenic [11]. Also, in a meta-analysis including 73 randomized trials comparing clinical outcomes in adult patients receiving colloids in the perioperative period, HES preparations were associated with a 15% reduction in blood loss compared to gelatin and pentastarches [11]. Crystalloids, on the other hand, are aqueous solutions of mineral salts or other water-soluble molecules. Although crystalloids come in a variety of formulations, a preparation like RL with an ionic composition close to that of plasma may be referred to as “balanced” or “physiological” [19]. The primary recognized difference between crystalloid and colloid solutions is the ability of colloid solutions to maintain or improve colloid osmotic pressure for longer periods in comparison with crystalloid solutions in which colloid osmotic pressure may be reliably reduced through hemodilution [20,21]. This is especially important in patients that have significant blood loss. Our study probably involved a very experienced surgical team that has caused minimal blood loss, and this could be the reason we did not found any differences in the two groups with respect to blood loss and the hemodynamic effects. However, as the amount of surgical blood loss increases, the maintenance of a normal colloid oncotic pressure may not be possible without the administration of a colloid solution. Even though crystalloids are theoretically associated with higher blood loss as compared to colloids, we observed that the blood loss was similar to RL and HES in our study.

The study had few limitations in terms of a small sample size and a study restricted to one indication and a single tertiary care center. Our study involved less intraoperative blood loss, but the hemodynamic parameters may be altered during moderate to severe blood loss, and the patients may require colloid or vasodilators. Hence, the observations could not be generalized to other surgical conditions and the entire population.

## Conclusions

Crystalloids may be a safe and cost-effective fluid replacement option during perioperative period in patients undergoing OP-CABG cardiac surgery in economically challenged countries. However, further large-scale multicenter studies with varied indications are recommended to substantiate the equivalence of crystalloids to colloids in perioperative management.
